# The *Non-Coding RNA* Journal Club: Highlights on Recent Papers—15

**DOI:** 10.3390/ncrna12040023

**Published:** 2026-07-17

**Authors:** Francisco J. Enguita, Suresh K. Alahari, Luca Agnelli, Raphaël Zemmour, Florent Hubé, Patrick K. T. Shiu, Sophia M. Zhang, Mohammadjavad Mohammadi, Shuxing Zhang, Sreyasree Dhar, Simon J. Conn, Agnieszka Bronisz, Jakub Godlewski, Abhishek Kaushik, Alexander Serganov, Barbara Pardini, André P. Gerber, Mark W. Feinberg, Eleonora Leucci, Phoebe Philpott, Andrea Caporali, Toshiaki Takahashi, Ajay Goel, Ling Yang

**Affiliations:** 1 Instituto de Genética, Faculdade de Medicina, Universidade de Lisboa, 1649-028 Lisbon, Portugal; 2 Department of Biochemistry and Molecular Biology, LSU School of Medicine, New Orleans, LA 70112, USA; 3 Department of Diagnostic Innovation, Fondazione IRCCS Istituto Nazionale dei Tumori, Via Venezian 1, 20133 Milan, Italy; 4 Développement Adaptation et Vieillissement (Dev2a), CNRS UMR8263, INSERM U1345, TErBio, IBPS, Sorbonne Université, 75005 Paris, France; raphael.zemmour.pro@gmail.com; 5 Division of Biological Sciences, University of Missouri, Columbia, MO 65211, USA; 6 Intelligent Molecular Discovery Laboratory (IMDLab), Cancer Biology Program, University of Hawaii Cancer Center, Honolulu, HI 96813, USA; 7 Flinders Health and Medical Research Institute, College of Medicine & Public Health, Flinders University, Bedford Park, SA 5042, Australia; dhar0071@flinders.edu.au; 8 Mossakowski Medical Research Institute, Polish Academy of Sciences, 02-106 Warsaw, Poland; jgodlewski@imdik.pan.pl; 9 Department of Biochemistry and Molecular Pharmacology, New York University Grossman School of Medicine, New York, NY 10016, USA; 10 Italian Institute for Genomic Medicine, Candiolo, 10060 Turin, Italy; 11 Discipline Microbes, Infection and Immunity, School of Biosciences, Faculty of Health and Medical Sciences, University of Surrey, Guildford GU2 7XH, UK; 12 Brigham and Women’s Hospital, Division of Cardiovascular Medicine, Heart & Vascular Institute, Harvard Medical School, Boston, MA 02115, USA; 13 Laboratory of RNA Cancer Biology, Department of Oncology, LKI, KU Leuven, 3000 Leuven, Belgium; 14 Institute of Neuroscience and Cardiovascular Research, University of Edinburgh, 47 Little France Crescent, Edinburgh EH16 4TJ, UK; p.g.philpott@sms.ed.ac.uk; 15 Department of Molecular Diagnostics and Experimental Therapeutics, Beckman Research Institute of City of Hope, Biomedical Research Center, Monrovia, CA 91010, USA; ttakahashi@coh.org; 16 Department of Gastroenterological Surgery, Okayama University Graduate School of Medicine, Dentistry, and Pharmaceutical Sciences, Okayama 700-8530, Japan; 17 City of Hope Comprehensive Cancer Center, Duarte, CA 91010, USA; 18 Department of Medical Genetics and Molecular Biochemistry, Lewis Katz School of Medicine at Temple University, Philadelphia, PA 19140, USA

## 1. Introduction

We are delighted to share with you our fifteenth Journal Club and highlight some of the most interesting papers published recently. We hope to keep you up-to-date with non-coding RNA research that extends beyond your study area. The *Non-Coding RNA* Scientific Board wishes you an exciting and fruitful read.

## 2. Too Hot to Self-Renew: HOTSCRAMBL lncRNA Rewires Human Hematopoietic Stem Cells

### Highlighted by Francisco J. Enguita

The study by Lyu and coworkers [1] identifies a previously uncharacterized long non-coding RNA (lncRNA), termed HOTSCRAMBL (HOXA opposite-strand transcript, stem-cell regulator, antisense mid-cluster between loci), as a key regulator of human hematopoietic stem cell (HSC) biology. Through genome-wide association and fine-mapping analyses, the authors identified the inherited variant rs17437411 within the HOXA locus as being associated with reduced blood cell counts, protection against clonal hematopoiesis and myeloid malignancies, and decreased leukocyte telomere length, suggesting reduced HSC self-renewal.

HOTSCRAMBL is highly enriched in primitive CD34^+^ hematopoietic stem/progenitor cells and localizes predominantly to the nucleus. Functional studies using CRISPR-Cas9 deletion and base-editing approaches demonstrated that the rs17437411 variant impairs long-term HSC maintenance by promoting proliferation and differentiation while reducing stem cell quiescence and self-renewal capacity both in vitro and in xenotransplantation models.

Mechanistically, HOTSCRAMBL regulates expression and co-transcriptional splicing of several HOXA genes, particularly HOXA9, a critical regulator of HSC identity and leukemogenesis. The rs17437411 variant alters the secondary structure of HOTSCRAMBL, weakening its interaction with the splicing factor SRSF2 and reducing recruitment of SRSF2 to HOXA9 pre-mRNA. This results in defective HOXA9 splicing, decreased HOXA9 mRNA and protein expression, and consequent loss of stem cell function.

Importantly, perturbation of HOTSCRAMBL also impaired growth and maintenance of HOXA-dependent acute myeloid leukemia (AML) models, linking this lncRNA to malignant hematopoiesis. Overall, the study reveals a previously unknown lncRNA-mediated mechanism controlling HOXA gene regulation and demonstrates how inherited non-coding genetic variation can fine-tune stem cell self-renewal while simultaneously protecting against hematologic malignancies.

## 3. Circulating microRNAs as a Framework for Comprehensive Early Diagnosis

### Highlighted by Suresh K. Alahari and Luca Agnelli

The conditions responsible for the “big non-oncological killers”, namely cardiovascular events, neurodegeneration, or pulmonary diseases, share a common and clinically inconvenient feature: their pathological substrate develops silently, years before overt symptoms require investigation. By the time a troponin rises or cognitive impairment becomes measurable, the disease can no longer be considered “early”, and reducing this diagnostic gaps remains among the most relevant needs in preventive medicine.

Blood-based molecular biomarkers have long been proposed as the solution for the aforementioned challenge. Among candidate species, extracellular circulating microRNAs (miRNAs) appeared to be good biomarkers to understand pathological changes in important organs: they are cell-type-specific, biochemically stable in biological fluids (owing to extracellular vesicle encapsulation), and notably sensitive to pathological change, which makes them ideal for diagnostic and eventually prognostic and monitoring purposes.

Wending Li and coauthors [2] aimed their study at translating circulating miRNA potential into a validated clinical framework. Their miRNA-based Tissue Signal (miR-TS) scores simultaneously assess 17 tissues (including the brain, heart, liver, and lung) and were robustly associated with relevant health outcomes across three population-based cohorts and 11 publicly available clinical populations with high tissue specificity. Specifically, the population cohorts include the U.S. Veteran affairs Normative Aging Study (NAS), the Shiyan Cohort from China, and the Dongfeng-Tongji cohort from China. Overall, from this study, authors were able to identify tissue-specific microRNA markers through the use of circulating microRNAs. In addition, they elegantly showed that the proposed miR-TS biomarkers prospectively tracked the development of acute myocardial infarction, cerebrovascular accidents, cognitive impairment, and airflow limitation. The potential clinical implications are immediate: a single blood draw might offer simultaneous multi-organ health surveillance, could redefine population screening, enable earlier intervention, and support individualized monitoring in chronic disease management with a low-cost impact. The validation across independent cohorts is encouraging, although the authors recognized some limitations (i.e., the limits in identifying specific cell-type origin within each tissue, the usage of relatively permissive false discovery rate, and the inability to define the timing of possible/predicted acute events): prospective interventional trials will therefore be essential to confirm that early detection of miR-TS signals can be easily translated into clinical practice, improving patients’ management and outcome.

## 4. Intronic Polyadenylation Is Necessary for Nucleolar Integrity and Activity

### Highlighted by Raphaël Zemmour and Florent Hubé

It is now known that more than half of eukaryotic genes have several Poly-Adenylation Sites (PASs) in their 3′UTR regions, which can give rise to slightly different transcripts (Alternative Poly-Adenylation, APA). Furthermore, around 20% of human genes also have PASs in their introns [3], which can result in shorter transcripts that are often considered aberrant and exhibit Intronic Poly-Adenylation (IPA).

In a recent article in *PNAS*, Mallick et al. [4] demonstrated the functional role of an IPA transcript, lncRNA CUL1-IPA, which modulates nucleolar integrity and function. Since the CUL1 gene is known to express Cullin, a protein of the SCF (SKP1-CUL1-F-box protein) ubiquitin ligase complex involved in the degradation of cell cycle progression proteins, CUL1-IPA appears to be the first demonstrated example of a functional IPA transcript with a completely different role than that of the protein encoded by the same gene. After verifying the polyadenylation of CUL1-IPA, its stability, its dependence on RNA polymerase II, and its expression, the authors investigated its role. Given that RNA localization is a determining factor in its function [4], the authors examined the localization of CUL1-IPA and observed that it is predominantly located in the nucleolus with nucleolar protein partners such as NOP58. In loss-of-function analysis of CUL1-IPA, the authors noted that nucleoli disintegrate, resulting in an increased number of smaller nucleoli. They also observed a reduction in the levels of rRNA (produced and matured in the nucleolus) and a reduction in overall protein synthesis, leading to cell cycle arrest in the G2/M phase. After confirming the role of CUL1-IPA with rescue experiments, the authors also noted that CUL1-IPA expression levels affect patient survival in certain types of cancer and that low expression of CUL1-IPA improves survival.

Overall, this work sheds light on the biological function of an lncRNA from a protein-coding gene and contributes to the credibility and importance of IPA transcripts.

## 5. A Trigger RNA Destroys a microRNA by Making Their Argonaute Carrier a Degradation Target

### Highlighted by Patrick K. T. Shiu

In eukaryotes, Argonaute (AGO) proteins can use microRNAs (miRNAs) to seek out complementary mRNAs for silencing. Although it has been established that miRNAs can be suppressed by unusual targets (called trigger RNAs) via a process known as target-directed miRNA degradation (TDMD), its underlying mechanism had not been elucidated until a recent study by Farnung and colleagues [5,6].

While a working TDMD model suggests that the binding of a trigger RNA to an AGO–miRNA complex recruits the ZSWIM8-CUL3 E3 ubiquitin ligase, the authors provided the first direct evidence that such a dual-RNA-occupied Argonaute is indeed bound by ZSWIM8 (the substrate receptor) and targeted for ubiquitylation. Cryogenic electron microscopy (cryo-EM) data showed that the miRNA–trigger pairing changes the conformation of their Argonaute carrier and promotes its binding to ZSWIM8. Interestingly, instead of recognizing a traditional ubiquitination signal (e.g., a peptide sequence or structural motif), the dimeric ZSWIM8 clamp interacts with two extended surfaces of the unusually folded Argonaute and the trigger RNA.

The results of this work help define a unique class of E3 ligases that mediate RNA-controlled protein decay. Future research in this area will hopefully provide insight into the prevalence of TDMD and its role in various cellular processes.

## 6. Toward an “AlphaFold Moment” for ncRNAs

### Highlighted by Shuxing Zhang

Predicting the three-dimensional (3D) structures of RNAs, especially ncRNAs, is a significant challenge in structural biology due to their high flexibility and the scarcity of experimental data, compared to proteins. In a recent issue of *Nature Machine Intelligence*, Wang and colleagues introduced trRosettaRNA2, a deep learning-based end-to-end algorithm that marks a major step toward overcoming these obstacles [7].

The core innovation of trRosettaRNA2 is its SS prior module (trRNA2-SS), which is pre-trained on extensive secondary structure (SS) data. Because 2D base-pairing information is more accessible than 3D coordinates, this module provides informative priors that guide the subsequent SS-aware structure module to generate accurate 3D conformers. In the CASP16 blind test, this approach was ranked on the top, even surpassing AlphaFold3. Remarkably, trRosettaRNA2 achieves this performance with only ~11 million parameters, roughly 1/33rd the size of AlphaFold3, demonstrating its computational efficiency.

For ncRNA research, the implications are profound. The authors applied trRNA2-SS to over 98,000 non-redundant ncRNA sequences that lacked known structures, providing high-confidence 2D and 3D structural references for various types, including lncRNAs, miRNAs, and ribozymes. From a drug discovery perspective, trRosettaRNA2’s ability to predict conformational ensembles is a game-changer. While methods like AlphaFold3 often produce static, homogeneous models, trRosettaRNA2 successfully captured the structural heterogeneity of RNase P RNA, matching experimental atomic force microscopy (AFM) data. This capability to explore the RNA conformational landscape is essential for identifying dynamic binding pockets, potentially accelerating the development of small molecules that target ncRNAs involved in diseases. This work provides a powerful framework for integrating deep learning with experimental probing to unlock the functional secrets of the non-coding genome.

## 7. Circle of Control: circMAN1A2 Drives CRC Through RNA and Protein Interactions

### Highlighted by Sreyasree Dhar and Simon J. Conn

Circular RNAs (circRNAs) are covalently closed, largely non-coding RNAs generated through back-splicing of precursor mRNAs. CircRNAs participate in diverse biological processes through their interactions with RNA, protein, and DNA [8]. Importantly, multiple circRNA isoforms can arise from a single gene locus through alternative circularization (AC), greatly expanding transcriptomic and functional complexity. However, despite the widespread occurrence of AC, the biological significance and functional diversity of individual circRNA isoforms remain poorly understood, particularly in cancer progression.

To address this gap, Cao et al. [9] systematically profiled the AC landscape across multiple human cell lines and colorectal cancer (CRC) tissues to identify predominantly expressed circRNAs (pe-circRNAs) with potential pathological relevance. Among these, *circMAN1A2(2,3,4,5)* emerged as a universally abundant and significantly upregulated circRNA in CRC. Functional analyses demonstrated that depletion of *circMAN1A2(2,3,4,5)* inhibited CRC cell proliferation, induced chromosome instability, and suppressed tumor growth in organoid and mouse heterotopic and orthotopic xenograft models.

Mechanistically, the study uncovered a highly novel regulatory mechanism in which *circMAN1A2(2,3,4,5)* directly interacts with the 3′ untranslated region (3′UTR) of CENPB mRNA through its unique back-splice junction (BSJ) sequence. Simultaneously, *circMAN1A2(2,3,4,5)* associates with the RNA-binding protein IGF2BP2, thereby enhancing IGF2BP2-mediated stabilization of CENPB mRNA, a mechanism consistent with the established role of IGF2BP proteins in regulating mRNA stability [10]. This circRNA-mRNA interaction preserves centromere integrity and promotes faithful chromosome segregation during cell division. Importantly, therapeutic targeting of the BSJ using locked nucleic acids selectively inhibited CRC progression with minimal effects on normal colorectal cells. Expanding this study to incorporate circRNA:mRNA interactions in non-cancerous cell lines would be a significant advance towards the therapeutic targeting of this phenomenon.

Overall, this work establishes a previously unrecognized paradigm in circRNA biology, demonstrating the critical role for circRNA-RNA-protein interactions to drive tumor progression. Further investigation may allow this phenomenon to be exploited to develop novel, targeted anticancer therapies.

## 8. From “Non-Coding” RNA to Peptides: Expanding the Human Proteome

### Highlighted by Agnieszka Bronisz and Jakub Godlewski

For many years, the dichotomy between “coding” and “non-coding” RNA implied a functional divide: some RNAs encode proteins, whereas others act through RNA-based mechanisms. A recent study from the TransCODE Consortium challenges this boundary and provides one of the most systematic views so far of translation from noncanonical open reading frames (ncORFs).

In a recent *Nature* study, Deutsch and colleagues analyzed 7264 ribosome-profiling-supported ncORFs and searched for peptide-level evidence across 95,520 proteomics entries. As much as 25% of these ncORFs produced detectable peptide-level evidence, with the bulk of this evidence coming from HLA immunopeptidomic datasets. This distinction is important: the study does not simply claim that thousands of ncRNAs encode canonical proteins. Instead, it shows that many translated ncORFs can enter the detectable peptide space, including the antigen-presentation pathway.

A major strength of the study is its cautious annotation framework. The authors distinguish among ribosome occupancy, peptide detection, evolutionary constraint, and functional evidence, and introduce the term “peptideins” for small translated products whose protein-like status is supported but whose biological function remains unclear. The study also moves beyond cataloging: functional analyses showed that a peptide encoded by the long non-coding RNA *OLMALINC* is broadly required for cell fitness, illustrating that at least some ncRNA-derived peptides have direct biological relevance.

By linking noncanonical translation to proteomic evidence and available annotation resources, this study provides a practical roadmap for deciding when an ncRNA-derived ORF is biologically meaningful. It also raises important questions for cancer biology, immunopeptidomics, and biomarker discovery, in which previously unannotated peptides may act as functional regulators, cancer-associated antigens, or markers of altered translation. Overall, this work does not diminish the regulatory importance of ncRNAs; instead, it expands their possible modes of action [11].

## 9. Small RNA-Based Polymerase

### Highlighted by Abhishek Kaushik and Alexander Serganov

The “RNA world” hypothesis proposes that life began with self-replicating RNA molecules (ribozymes). However, previously identified “polymerase” ribozymes were large (>150 nucleotides) and complex, making it unlikely they emerged spontaneously on early Earth. The authors of a recent study hypothesized that a much smaller, simpler RNA motif could catalyze RNA polymerization under certain conditions [12]. To find the shortest possible motif, they performed in vitro directed evolution from a randomized RNA pool in eutectic ice, a medium known to stabilize polymerase ribozymes and concentrate substrates [13]. After mutagenesis and truncations, they found that a 45-nucleotide version, called QT45, retained nearly all the original polymerase activity and a dense catalytic core. This ribozyme uses a primer and activated trinucleotide "triplets" as building blocks for RNA synthesis and can copy various RNA templates, including those with stem-loops and tightly folded secondary structures. QT45 most efficiently incorporates trinucleotide blocks but can also accept longer oligonucleotides, supporting the complex mixture of oligonucleotide blocks in the prebiotic environment. QT45 can operate in trans, acting on a separate primer-template complex without being physically attached, much like larger ribozymes. Most importantly, QT45 is capable of self-replication through two key steps: first, synthesizing the (−) strand under optimized conditions, and second, synthesizing the (+) strand using triplet nucleotide blocks. Despite functioning as a true RNA polymerase, the ribozyme requires specialized conditions, with a slow overall reaction rate and low yield. Nonetheless, this discovery highlights another non-coding capability of RNA and strongly supports the RNA world hypothesis.

## 10. SNORD105B Is a Novel Biomarker of Chronic Kidney Disease Risk and SGLT2 Inhibitor Response in Type 2 Diabetes

### Highlighted by Barbara Pardini

The biological role of many small non-coding RNAs (sncRNAs) remains poorly understood, and several classes, including small nucleolar RNAs (snoRNAs), have received considerably less attention than microRNAs (miRNAs). Previous studies have shown that miRNAs transported through the bloodstream can influence the fate and function of recipient cells and tissues, including kidney cells. Whether similar roles are played by other classes of circulating sncRNAs remains largely unexplored.

In the study by de Klerk and colleagues, the authors investigated the association between circulating sncRNAs and the development of chronic kidney disease (CKD), a common complication of type 2 diabetes, as well as their response to sodium-glucose cotransporter-2 (SGLT2) inhibitor treatment. Plasma sncRNA profiles were analyzed in 263 individuals with type 2 diabetes who were free of CKD at baseline and followed for more than nine years. In addition, circulating sncRNAs were profiled before and after SGLT2 inhibitor treatment in independent clinical cohorts [14].

Among the eleven sncRNAs associated with incident CKD, the snoRNAs *SNORD12C* and *SNORD105B* showed the strongest associations. Notably, *SNORD105B* was also among the sncRNAs whose expression changed following SGLT2 inhibitor treatment. Although these findings require validation and most associations did not remain significant after full adjustment or multiple-testing correction, the study highlights *SNORD105B* as a promising candidate biomarker of CKD risk and therapeutic response. Furthermore, correlations between *SNORD105B* and circulating proteins suggest a potential role in extracellular signaling pathways, providing a rationale for future functional studies.

Overall, this work supports the concept that sncRNA classes beyond miRNAs may act as mediators of inter-organ communication and contribute to the maintenance of kidney function. While the biological functions of many circulating snoRNAs remain poorly characterized, the study by de Klerk and colleagues provides additional evidence that these molecules may have previously unrecognized roles in diabetic kidney disease and deserve further mechanistic investigation.

## 11. Human lncRNA *RMRP* as a Mg^2+^-Responsive RNA Scaffold Linking Helicase Activity, Localization and Mitochondrial Function

### Highlighted by André P. Gerber

*RMRP* is an evolutionarily conserved lncRNA that acts as the RNA component of the RNase MRP ribonucleoprotein complex. RNase MRP has established roles in mitochondrial RNA processing, pre-rRNA maturation, and cell-cycle regulation, and mutations in the *RMRP* gene cause cartilage-hair hypoplasia, a rare developmental and immunological disorder. However, despite its biological importance, the structural dynamics of *RMRP* and how they relate to its cellular functions have remained incompletely understood.

Pereira and colleagues provided a conceptually interesting view of *RMRP* as a dynamic regulatory RNA rather than a static RNP component [15]. Using small-angle X-ray scattering, they showed that *RMRP* can adopt different conformations depending on Mg^2+^ concentration, suggesting that divalent cations may influence its higher-order folding and raising the intriguing possibility that *RMRP* could behave as a Mg^2+^-responsive RNA scaffold. RNA pull-down experiments further identified the DEAD-box helicases DDX5 and DDX3X as *RMRP*-associated proteins. These interactions were supported by biochemical assays showing direct binding, with DDX5 displaying stronger binding and ATP-dependent helicase activity on *RMRP*. Interestingly, knockdown of *DDX5* or *DDX3X* in PC-3M prostate cancer cells, where *RMRP* is highly expressed, reduced mitochondrial localization of *RMRP*. In turn, *RMRP* knockdown affected mitochondrial membrane potential, ROS production, the expression of selected nuclear-encoded mitochondrial transcripts, and oxidative phosphorylation complex assembly, particularly complex IV. Surprisingly, *RMRP* depletion also lowered mitochondrial free Mg^2+^ levels, suggesting possible feedback between *RMRP* structure, its function, and mitochondrial Mg^2+^ homeostasis.

Overall, the work adds an interesting conceptual link between RNA structure, helicase-mediated remodeling, and organelle physiology. Rather than acting only as a component of a canonical processing enzyme, *RMRP* may represent a structurally adaptable lncRNA that relays changes in the cellular environment to mitochondrial regulatory outputs. In this view, lncRNAs are not merely passive scaffolds for protein complexes, but dynamic molecular devices whose folding, localization, and protein interactions contribute to cellular homeostasis.

## 12. Turning on the CHARM in Cardiomyopathy

### Highlighted by Mark W. Feinberg

While it was initially thought that most long non-coding RNAs (lncRNAs) were rarely conserved across species [16,17,18], accumulating studies highlight a growing list of lncRNAs that exhibit higher levels of homology and concordant cellular functional responses [19,20,21]. The study by Buonaiuto et al. [22] identifies and characterizes the human ortholog of the mouse cardiac-enriched lncRNA CHARME, termed HSCHARME. Using comparative genomics, single-cell transcriptomics, RNA imaging, CRISPR/Cas9 genome editing, and human-induced pluripotent stem cell-derived cardiomyocytes (hiPSC-CMs), the authors demonstrate its important role in human cardiomyocyte differentiation, function, and cardiomyopathy.

The authors found that the predominant transcript produced from the HSCHARME locus is pCHARME, which is highly expressed in human atrial and ventricular cardiomyocytes. pCHARME localizes near SC35-positive nuclear speckles and physically interacts with the RNA-binding protein PTBP1, a key regulator of alternative splicing. Through this interaction, pCHARME helps coordinate the splicing of numerous cardiac-specific pre-mRNAs that are essential for cardiomyocyte identity and function.

Deletion of pCHARME in hiPSC-derived cardiomyocytes generated extensive defects in cardiomyogenesis, including altered alternative splicing programs, reduced expression of genes involved in cardiac differentiation, and impaired acquisition of mature cardiomyocyte features. Cells lacking pCHARME displayed delayed onset of spontaneous beating, reduced contraction frequency, abnormal morphology, and markedly decreased cardiac progenitor cells and mature cardiomyocytes. Knockdown experiments in already differentiated cardiomyocytes suggested that pCHARME is required for cardiomyocyte specification and the maintenance of the differentiated state. The authors found that pCHARME expression, but not the fully spliced mature transcript mCHARME, is significantly elevated in both hypertrophic cardiomyopathy and dilated cardiomyopathy.

Taken together, the study establishes HSCHARME as a conserved regulator of human cardiac gene splicing and differentiation and implicates pCHARME dysregulation in human cardiomyopathy. Future studies will need to reconcile whether the elevated expression of pCHARME is a compensatory, protective mechanism in response to myocardial injury and its broader utility in genetic cardiomyopathy.

## 13. A Pathogen lncRNA Secreted into Rice Sequesters a Host miRNA for Virulence

### Highlighted by Eleonora Leucci

This study identifies a novel cross-kingdom virulence mechanism in which the rice blast fungus *Magnaporthe oryzae* secretes a long non-coding RNA (lnc117761) into host rice cells to suppress immunity. Using RNA seq and mutant analysis, the authors show that lnc117761 is highly expressed during infection and is required for fungal pathogenicity. Through transgenic rice lines and CRISPR knockouts, they demonstrate that a host microRNA, miR5827, positively regulates disease resistance by repressing the immune negative kinase gene PKR1. Biochemical assays (EMSA, ITC, RNA pull down, and luciferase reporters) confirm that lnc117761 directly binds miR5827 via a complementary sequence, acting as a “sponge” to reduce its availability. Deletion and mutation of the miRNA binding site abolish both binding and virulence, proving functional relevance. Microscopy, in situ hybridization, and extracellular vesicle isolation show that lnc117761 is secreted by the fungus and translocated into infected and neighboring plant cells. Functional assays (overexpression, knockout, and infection studies) establish that sequestration of miR5827 increases PKR1 expression and weakens host immunity, facilitating infection. Comparative sequence and functional analyses across multiple pathogens and plants reveal that this RNA–RNA interaction motif is evolutionarily conserved. Finally, application of synthetic miRNA mimics enhances disease resistance in rice and wheat, demonstrating potential for crop protection. Together, the data establish a new paradigm of RNA–RNA-mediated host–pathogen interaction driven by a secreted fungal lncRNA [23].

## 14. Inhibiting MicroRNA-132 in Adverse Cardiac Remodeling: Potential Implications for miRNA-Based Clinical Trials

### Highlighted by Phoebe Philpott and Andrea Caporali

miR-132 increases with myocardial stress, leading to adverse remodeling and heart failure. Preclinical studies demonstrated that inhibition of miR-132 can effectively reverse cardiac dysfunction across various heart failure models [24] and in a phase I trial [25].

In this study, the authors carried out a phase II trial called HF-REVERT to evaluate CDR132L, an antisense oligonucleotide targeting miR-132, in patients after myocardial infarction (MI) with left ventricular (LV) systolic dysfunction [26]. The trial included 294 patients with a left ventricular ejection fraction (LVEF) of 45% or less shortly after MI, who were randomized to receive either 5 mg/kg or 10 mg/kg of CDR132L or placebo, alongside standard post-MI treatment. The main goal was to measure the percentage change in left ventricular end-systolic volume index (LVESVI) at six months, a recognized indicator of ventricular remodeling [26]. Despite dose-dependent suppression of circulating miR-132, confirming target engagement, CDR132L did not meet its primary endpoint, showing no significant improvement in LV remodeling compared with placebo. Secondary outcomes, like ejection fraction, global longitudinal strain, and NT-proBNP, were also unchanged. Although it did not meet its primary endpoint, the study demonstrates that miR-132-based therapy is feasible in heart failure.

The lack of clinical significance likely reflects limitations of the trial design, not biology. The rebound in miR-132 levels after treatment cessation suggests that longer exposure may be needed and that structural remodeling may take more than six months to become visible [27]. The widespread use of SGLT2 inhibitors as background therapy may have lowered residual modifiable risk. Many participants had limited baseline remodeling, which may have masked treatment effects. Women were underrepresented (13%), a limitation given sex differences in remodeling biology [28].

Overall, these results emphasize the need to improve patient selection and treatment timing in future clinical trials on miRNA-based therapies for cardiovascular disease.

## 15. MicroRNA-25 Drives Immune Checkpoint Therapy Resistance Through Remodeling of the Tumor Microenvironment

### Highlighted by Toshiaki Takahashi and Ajay Goel

Immune checkpoint therapy (ICT) has transformed the treatment landscape for several malignancies, leading to durable clinical responses in a subset of patients. However, intrinsic and acquired resistance to ICT remains a major clinical challenge, underscoring the need to elucidate the molecular mechanisms that enable tumor immune evasion.

In a recent study, Zhu et al. identified microRNA-25 (miR-25) as a critical regulator of ICT resistance. Using melanoma, colorectal cancer, and breast cancer models, the authors demonstrated that genetic deletion of miR-25 significantly enhanced responsiveness to anti-PD-1 therapy. Notably, loss of miR-25 had minimal effects on tumor cell proliferation, suggesting that its primary role is mediated through remodeling of the tumor microenvironment rather than direct regulation of tumor growth [29].

Single-cell transcriptomic analyses revealed that miR-25 deficiency increased the abundance of antigen-presenting macrophages and enhanced complement-associated inflammatory signaling in cancer-associated fibroblasts. These changes promoted both innate and humoral immune responses, creating a more inflammatory tumor microenvironment that was conducive to effective immunotherapy. Importantly, these effects were consistently observed across multiple tumor models, suggesting that the underlying mechanism may be broadly applicable across diverse cancer types.

Mechanistically, the authors identified Syndecan-3 (SDC3) as a direct target of miR-25. In response to interferon-γ signaling, miR-25 suppresses SDC3 expression, thereby attenuating antitumor immunity. Restoration of SDC3 expression, either through miR-25 deletion or by disrupting the miR-25 binding site within the SDC3 3′ untranslated region, enhanced sensitivity to immune checkpoint blockade. These findings establish the miR-25–SDC3 axis as a key regulator of the dynamic crosstalk between tumor cells and the surrounding immune microenvironment.

Collectively, this study uncovers a previously unrecognized mechanism of immune resistance mediated by a non-coding RNA. Beyond providing important mechanistic insights into tumor immune evasion, the findings highlight the therapeutic potential of targeting miR-25 to remodel the tumor microenvironment and enhance responses to immunotherapy. The miR-25–SDC3 axis may therefore serve not only as a predictive biomarker of ICT response but also as a promising therapeutic target for overcoming resistance to immune checkpoint inhibitors.

## 16. A New lncRNA Target for Obesity Therapy

### Highlighted by Ling Yang

Obesity affects more than one billion people worldwide, highlighting an urgent need for novel therapeutic targets. Emerging evidence indicates that long non-coding RNAs (lncRNAs) hold significant promise as therapeutic targets for managing obesity. Notably, a recent original article by Abdollahi et al., published in *Molecular Therapy-Nucleic Acids*, identified a new lncRNA as a potential therapeutic target [30].

In this study, the authors discovered the detrimental effects of the lncRNA lnc-megacluster (lncMGC) in both white and brown adipose tissues. Targeting lncMGC using CRISPR-based knockout or GapmeR antisense oligonucleotides prevents high-fat diet (HFD)-induced obesity and adipose tissue dysfunction in mice. In addition, to assess translational relevance, the authors utilized partially humanized lncMGC mice to demonstrate that GapmeRs specifically targeting the human lncMGC sequence effectively mitigate high-fat diet-induced obesity. Mechanistically, they demonstrated that lncMGC functions by modulating ER stress, adipogenesis, thermogenesis, and mitochondrial function in adipose tissues.

Taken together, this study suggests that lncMGC is a promising therapeutic target for treating obesity and its associated complications.
